# Atomic-Scale Molecular Dynamics Modeling of Iron Oxides: Surface Properties and Methodologies

**DOI:** 10.3390/molecules31101629

**Published:** 2026-05-12

**Authors:** Nikoleta Ivanova, Hassan Chamati

**Affiliations:** 1 Department of Physical Chemistry, University of Chemical Technology and Metallurgy, 8 Kliment Ohridski Blvd., 1756 Sofia, Bulgaria; n.ivanova@uctm.edu; 2 Institute of Solid State Physics, Bulgarian Academy of Sciences, 72 Tzarigradsko Chaussée, 1784 Sofia, Bulgaria; 3 National Centre of Excellence Mechatronics and Clean Technologies, 8 Kliment Ohridski Blvd., Blk. 8, 1756 Sofia, Bulgaria

**Keywords:** hematite, magnetite, maghemite, molecular dynamics, surface chemistry

## Abstract

Iron oxides, including hematite (α-${\mathrm{Fe}}_{2}$$O_{3}$), magnetite (${\mathrm{Fe}}_{3}$$O_{4}$), and maghemite (γ-${\mathrm{Fe}}_{2}$$O_{3}$), play central roles in catalysis, corrosion, environmental remediation, magnetic nanotechnology, and energy storage. Molecular dynamics simulations have become an essential tool for understanding their structural, magnetic, and interfacial behavior at the atomic scale. This review provides a comprehensive overview of MD methodologies applied to these materials, spanning classical force fields, reactive force fields, ab initio molecular dynamics, and emerging machine learning interatomic potentials. Particular emphasis is placed on facet-dependent surface chemistry, especially the contrast between compact (111) and open (110) planes, and on adsorption processes involving water, nitrogen-containing molecules, and representative organic compounds. The review highlights recent advances in force field development, redox modeling, and multiscale simulation strategies while critically identifying limitations related to charge transfer, mixed valence, vacancy ordering, and magnetic–chemical coupling. Finally, future perspectives are outlined toward quantitatively predictive, facet-resolved, and magnetically aware simulations of iron oxide interfaces. These developments are expected to tightly link atomistic insights with experimental observations and guide the rational design of iron oxide-based functional materials.

## 1. Introduction

Iron oxides are among the most abundant and versatile metal oxides, exhibiting unique chemical, electronic, and magnetic properties that establish them as integral to a wide range of technological and environmental applications. Some of the compounds that have attracted considerable interest are hematite (α-${\mathrm{Fe}}_{2}$$O_{3}$), magnetite (${\mathrm{Fe}}_{3}$$O_{4}$), and maghemite (γ-${\mathrm{Fe}}_{2}$$O_{3}$), each with distinctive crystallographic structures, electronic configurations, and surface reactivity patterns [1]. These oxides are used extensively in catalysis, such as CO oxidation, Fischer–Tropsch synthesis, and water splitting, due to their ability to facilitate surface electron transfer at active sites [2,3]. Recent reviews also highlight the continued relevance of ${\mathrm{Fe}}_{3}$$O_{4}$-based materials in catalytic applications and of ${\mathrm{Fe}}_{2}$$O_{3}$ among binary oxide ceramics for solar cell technologies [4,5]. Additionally, they are key reactants in environmental remediation, effectively adsorbing heavy metals and organic pollutants [6], and in energy storage, functioning as electrode materials in lithium-ion and sodium-ion batteries [7,8]. In biomedical and magnetic applications, iron oxides act as MRI contrast agents, magnetic hyperthermia mediators, and drug delivery vehicles, highlighting their multifunctional potential [9,10,11,12]. Recent reviews on ${\mathrm{Fe}}_{3}$$O_{4}$ and ${\mathrm{Fe}}_{3}$$O_{4}$-based nanocomposites further emphasize their current relevance in wastewater treatment, catalysis, green energy production, and biomedical applications [13].

A fundamental feature underlying the performance of iron oxides is their surface orientation, specifically the crystallographic surfaces taking part in the chemical reactions. The (111) and (110) planes of hematite, magnetite, and maghemite display significant dissimilarities in coordination, surface energy, and chemical reactivity [14]. The (111) plane tends to be more stable and hydroxylated, favoring molecular adsorption and weaker reactivity, while the (110) plane is more open (lower packing density), with under-coordinated Fe and O atoms that enhance adsorption, surface reconstruction, and catalytic activity. These plane-specific characteristics influence not only adsorption energies but also reactive processes, proton transfer, hydrogen bond network formation, and magnetic interactions, underscoring the necessity of detailed atomic-level investigations [14].

Atomistic insights into the structural, thermal, and reactive behavior can be probed with the aid of molecular dynamics (MD). Among others, MD facilitates studies of lattice dynamics, magnetic properties, phase transitions, surface reactions, and oxidation processes that are hardly accessible experimentally or using static electronic structure methods [3,15]. Moreover, MD allows explicit modeling of thermal motion, molecular adsorption, surface reconstruction, and dynamic interactions with solvents and adsorbates.

MD simulations are extensively used to gain useful insights on atomistic processes occurring at a specific sample surface. In catalysis, MD helps understanding adsorption processes and reactive pathways, thus providing useful information about the surface that favor selective reactions. For environmental remediation, MD predicts how water and pollutant molecules interact with iron oxides surfaces [16,17]. In energy storage and sensors, the adsorption of small molecules affects surface reactivity and transport, influencing device performance. Moreover, the atomic-scale features unveiled by MD boost effective defect engineering, vacancy optimization, and functionalization strategies, particularly for the (110) plane, which exhibit higher reactivity [14,18]. MD simulations have demonstrated that these plane-specific features regulate the behavior of water, nitrogen-containing molecules (e.g., ${\mathrm{NH}}_{3}$, $N_{2}$$H_{4}$, amines), and small organics, providing predictive insight into surface chemistry [19,20].

MD simulations not only complement experimental studies but also foster the design of iron oxide materials with tailored reactivity and selectivity properties, particularly by exploiting the distinct features of (111) and (110) surfaces [14,21]. The main challenges in MD simulations of iron oxides are the suitable force field selection and the accurate representation of chemical reactions (bond breaking and formation).

This review provides a comprehensive overview of atomistic-level insights into a class of iron oxides. It concentrates mainly on MD-based atomistic modeling that explores the physical and chemical properties of iron oxides by calculating atomic interactions and positions. Other aspects, such as electronic-structure properties computed via DFT and DFT+*U* methods, are discussed primarily to provide reference data, parameterization targets, validation benchmarks, or mechanistic context for MD simulations. The review begins by introducing the crystal structure of iron oxides, highlighting key surface terminations and structural features relevant to reactivity. Then, it examines MD methodologies used to model these materials, including classical force fields, reactive force fields, ab initio MD, and emerging machine learning interatomic potentials. Furthermore, the review focuses on the adsorption of small molecules on iron oxide surfaces, emphasizing water, nitrogen-containing molecules and some organic molecules, along with the governing interaction mechanisms. Finally, it deals with open challenges and future perspectives, addressing limitations in current modeling approaches and outlining directions for advancing predictive simulations of iron oxide surfaces.

## 2. Crystalline Structure of Iron Oxides

The crystalline structures of iron oxides are fundamental in shaping their surface properties, adsorption behavior, and catalytic potential. In Table 1, we summarize the main structural properties of hematite, magnetite, and maghemite. Further, we discuss their implications for surface chemistry and adsorption phenomena.

### 2.1. Hematite (α-Fe_2_O_3_)

Hematite (α-${\mathrm{Fe}}_{2}$$O_{3}$) adopts a corundum-type crystal structure with space group R$\bar{3}c$, characterized by oxygen anions in a hexagonal close-packed arrangement with iron cations occupying two thirds of the octahedral interstices [22,23]. In this arrangement, each ${\mathrm{Fe}}^{3+}$ ion is coordinated to six $O^{2-}$ ions to form edge-sharing Fe$O_{6}$ octahedra, connected along the [0001] direction. The lattice parameters at ambient conditions are approximately a=b≈5.04 Å and c≈13.75 Å [1]. The Fe-O bond lengths within the octahedra are not identical due to trigonal distortion, typically ranging between ∼1.94 and ∼2.11 Å, influencing both the electronic band structure and magnetic ordering [23]. Each ${\mathrm{Fe}}^{3+}$ has a $3d^{5}$ electronic configuration in a high-spin state, and the octahedral crystal field splits the 3d orbitals into lower-energy $t_{2g}$ and higher-energy $e_{g}$ orbitals [24]. The oxygen 2p orbitals interact with Fe 3d orbitals, forming bonding and antibonding states that determine the electronic band structure and semiconducting behavior of hematite [1].

**Figure 1:**
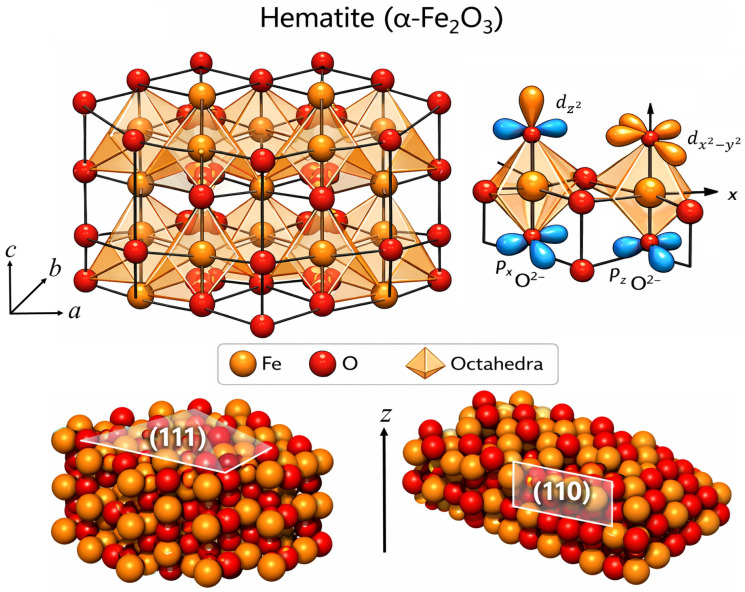
Unit cells of hematite (α-${\mathrm{Fe}}_{2}$$O_{3}$) with Fe 3d and O 2p atomic orbitals; two representative crystallographic planes (111) and (110). Crystallographic axes and surface orientations are indicated in the figure.

The crystal structure of α-${\mathrm{Fe}}_{2}$$O_{3}$ (hematite), including the basal plane, is shown in Figure 1. In the hexagonal setting, this surface is commonly denoted using Miller–Bravais four-index notation as (0001); however, for consistency, we use three-index Miller notation to comply with MD and DFT studies throughout the review, while the corresponding basal orientation is denoted as (111).

At the (111) planes, Fe ions are fully coordinated by oxygen, resulting in a relatively uniform electron density and stable electronic configuration at the surface. In contrast, undercoordinated Fe and O atoms at the (110) plane lead to localized states and higher electron density variations, which can influence surface redox activity and adsorption processes [14,22]. These plane-specific differences highlight the role of coordination and crystal field effects in modulating the electronic distribution. The (110) plane, orthogonal to the basal direction, exhibits a different arrangement of Fe and O coordination, with alternating Fe-O-O layers. These structural distinctions give rise to disparate electronic densities and coordination environments at the surface, which critically influence adsorption thermodynamics and reaction pathways. The (111) plane of hematite in hexagonal indexing reveals alternating layers of iron and oxygen atoms normal to the *c*-axis and is the most extensively studied facet in catalysis and photoelectrochemical research [22]. Reconstructions and terminations depend sensitively on oxygen chemical potential and preparation conditions, leading to bulk-like, oxygen-terminated, or reduced bi-phase surfaces [22].

Theoretical surface energy analyses indicate that the (110) plane has lower surface broken bond density and surface energy relative to higher-energy orientations such as (111), making it one of the most favorable facets in certain growth environments [25]. Ab initio MD (AIMD) enables spin-polarized calculations within DFT, revealing the hybridization between Fe 3d and O 2p orbitals as well as antiferromagnetic spin coupling that governs the local electronic density and band structure [26].

At the atomic scale, differences in coordination and electronic structure between low-index planes determine the surface chemical behavior. Surfaces with high coordination (e.g., (111) basal planes) tend to be relatively stable and favor molecular physisorption, whereas more open surfaces like (110) present undercoordinated Fe sites that facilitate stronger chemisorption and reactive dissociation pathways [25,27]. Detailed DFT investigations of low-index hematite surfaces have shown that surface energies and broken bond densities vary systematically among planes, with (110) generally having lower surface energy than (111), in agreement with experimental observations of facet exposure in nanoparticles and thin films [25,28]. Deviations from this electronic distribution occur at surfaces, edges, and defect sites, where undercoordination and symmetry breaking introduce mid-gap or surface states that strongly influence electron transfer processes, surface reactivity, and magnetic properties at the atomic scale [29].

### 2.2. Magnetite (${\mathrm{Fe}}_{3}$$O_{4}$)

Magnetite (${\mathrm{Fe}}_{3}$$O_{4}$) crystallizes in the inverse spinel structure with space group $Fd\bar{3}m$, where oxygen anions form a face-centered cubic lattice and iron cations occupy both tetrahedral (${\mathrm{Fe}}_{A}^{3+}$) and octahedral (${\mathrm{Fe}}^{2+}/{\mathrm{Fe}}_{B}^{3+}$) interstitial sites. A typical experimental lattice parameter is a=b=c≈8.396 Å at room temperature ($\alpha =\beta =\gamma =90^{\circ }$), with slight variations depending on temperature, stoichiometry, and defect concentration [1]. The electronic structure is characterized by mixed valence at the octahedral B sites and ferrimagnetic ordering arising from antiferromagnetic coupling between A and B sublattices [30]. Spin-polarized density functional theory (DFT) calculations show that magnetite exhibits half-metallic behavior in its high-temperature cubic phase, with minority-spin Fe 3d states crossing the Fermi level while majority-spin states remain insulating [31]. The electron distribution is therefore strongly spin-dependent, with B-site Fe 3d orbitals dominating the states near the Fermi energy and mediating electron hopping between ${\mathrm{Fe}}^{2+}$ and ${\mathrm{Fe}}^{3+}$ centers.

**Figure 2:**
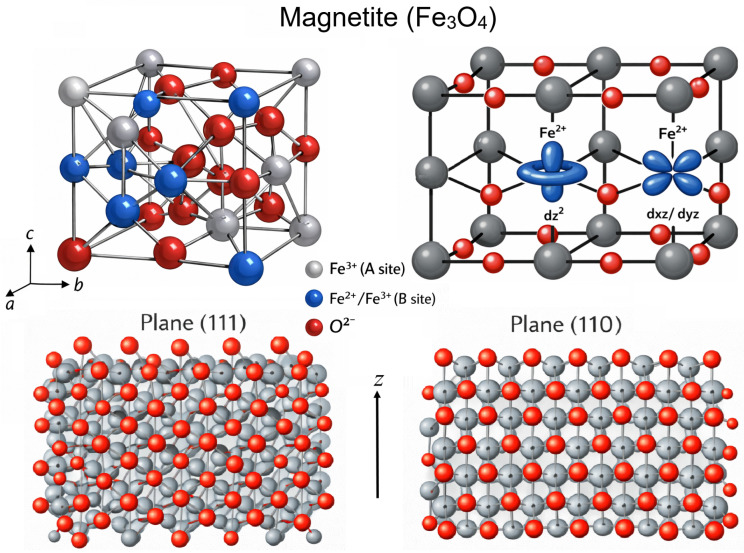
The crystal structure of magnetite (${\mathrm{Fe}}_{3}$$O_{4}$) with tetrahedral (A) and octahedral (B) cation sites; atomic 3d orbitals of ${\mathrm{Fe}}^{2+}$; the crystallographic (111) and (110) planes. Crystallographic axes and surface orientations are indicated in the figure.

The crystal structure of magnetite (${\mathrm{Fe}}_{3}$$O_{4}$) in the inverse spinel configuration, with tetrahedral (A) and octahedral (B) cation sites indicated, is shown on Figure 2. The atomic 3d orbitals of ${\mathrm{Fe}}^{2+}$ ions are illustrated to highlight their electronic configuration within the octahedral environment, and the (111) and (110) surfaces are also depicted, emphasizing their distinct atomic arrangements and coordination geometries.

The polar (111) surface consists of alternating close-packed oxygen and iron layers along the 〈111〉 direction, with several terminations possible depending on the oxygen chemical potential [32]. DFT+*U* calculations, commonly used to account for strong electronic correlations in iron oxides, indicate that surface stabilization can involve relaxation and charge redistribution [33], although hybrid functionals and other electronic-structure approaches may also provide realistic descriptions of these materials. Fe-terminated surfaces exhibit prominent ${\mathrm{Fe}}_{B}$ 3d minority-spin states near the Fermi level, enhancing metallicity, whereas oxygen-terminated surfaces have increased O 2p character and reduced Fe 3d weight, reflecting polarity compensation [34]. In contrast, the nonpolar (110) surface displays alternating rows of octahedral Fe and O atoms, leading to anisotropic coordination. Relaxation and B-site electron redistribution modify the density of states and local magnetic moments, influencing surface stability and adsorption behavior [34].

A comparison of the projected density of states (PDOS) between (111) and (110) surfaces highlights the facet-dependent electronic reconstruction. While bulk ${\mathrm{Fe}}_{3}$$O_{4}$ is half-metallic, surface symmetry breaking modifies spin polarization and Fe 3d bandwidths [35]. On Fe-terminated (111) surfaces, minority-spin ${\mathrm{Fe}}_{B}$ 3d states remain at the Fermi level, whereas oxygen termination suppresses these states due to charge transfer toward surface O atoms. The (110) surface shows anisotropic splitting of ${\mathrm{Fe}}_{B}$ $t_{2g}$ and $e_{g}$ states and partial localization of minority-spin electrons, reducing surface spin polarization. Broken Fe-O bonds shift the Fe 3d center of gravity to lower energies, affecting adsorption and catalytic activity. These plane-dependent electronic features illustrate the strong coupling between crystallographic orientation, coordination, and interfacial reactivity.

### 2.3. Maghemite (γ-${\mathrm{Fe}}_{2}$$O_{3}$)

**Figure 3:**
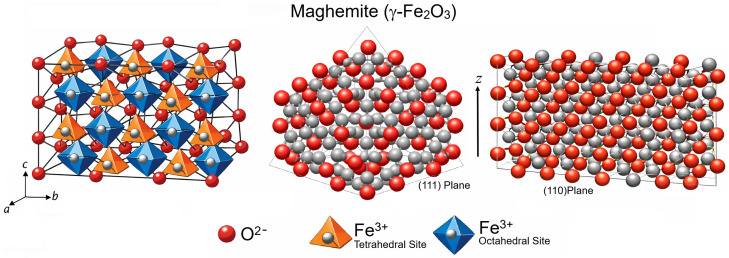
Crystal structure of maghemite (γ-${\mathrm{Fe}}_{2}$$O_{3}$) with tetrahedral and octahedral cation sites; the crystallographic (111) and (110) planes. Crystallographic axes and surface orientations are indicated in the figure.

Maghemite (γ-${\mathrm{Fe}}_{2}$$O_{3}$) is commonly described as a *defective inverse spinel* in which $O^{2-}$ anions form a cubic close-packed (fcc) lattice and Fe cations occupy both tetrahedral (A) and octahedral (B) interstices. In contrast to magnetite, all iron ions are formally ${\mathrm{Fe}}^{3+}$, and charge neutrality is achieved by introducing cation vacancies predominantly on the octahedral sublattice. The resulting vacancy order can lower the symmetry relative to the parent $Fd\bar{3}m$ spinel (often treated as cubic on average, with partially ordered vacancy patterns depending on synthesis and thermal history). A representative lattice parameter is a=b=c≈8.33–8.35 Å at room temperature ($\alpha =\beta =\gamma =90^{\circ }$), varying depending on the arrangement of vacancies and structural disorder [1]. A DFT+*U* has shown that vacancy ordering is not a minor perturbation, as it controls the distribution of local Fe-O coordination environments, stabilizes the ferrimagnetic A-B sublattice alignment, and shapes the band edges through spin-dependent Fe 3d–O 2p hybridization [36]. In the bulk-like electronic picture, the upper valence band is dominated by a O 2p character hybridized with Fe 3d, while the conduction-band onset largely stems from Fe 3d, with the band gap and near-edge spin polarization being sensitive to the vacancy configuration and the Hubbard correction [36]. The crystal structure of maghemite (γ-${\mathrm{Fe}}_{2}$$O_{3}$) illustrating the spinel and the crystallographic (111) and (110) planes is shown on Figure 3. The (111) plane corresponds to the close-packed oxygen stacking direction, whereas the (110) plane represents a more open surface with distinct Fe coordination environments.

For the polar (111) orientation, maghemite exhibits a close-packed O/Fe stacking sequences in which the *effective* surface charge and dipole depend on the specific termination (${\mathrm{Fe}}_{\mathrm{tet}}$-rich vs. ${\mathrm{Fe}}_{\mathrm{oct}}$-rich vs. O-rich) and on how subsurface vacancies are arranged. Recent periodic DFT+*U* slab calculations [37,38] that explicitly compare multiple (111) terminations report substantial structural relaxation and a systematic shrinking of the surface band gap relative to the bulk, alongside a redistribution of PDOS near the band edges. In these models, the valence-band maximum remains largely O 2p-derived, whereas the conduction-band minimum retains strong Fe 3d weight, but surface symmetry breaking enhances the contribution of undercoordinated surface Fe (especially octahedral-like sites) to the low-lying unoccupied states. This has a direct chemical implication: adsorption events that donate electron density (Lewis bases) preferentially couple into Fe-centered acceptor levels near the conduction edge, while oxidizing adsorbates (electron acceptors) couple strongly to the upper O 2p manifold, thereby tuning interfacial charge transfer on (111) [37,38].

Compared with (111), the (110) plane of defective spinels is structurally more open, displaying rows of coordinatively unsaturated Fe-O motifs and a higher density of low-symmetry ligand fields. While systematic *surface-resolved* DFT+*U* PDOS datasets for maghemite (110) remain much less common than for (111) in the recent literature, the same defect physics implies a clear qualitative expectation: (110) exhibits stronger facet-induced crystal-field splittings of surface Fe 3d ($t_{2g}/e_{g}$-like) manifolds and a higher propensity for localized surface (and vacancy-associated) states within the bulk gap, particularly when octahedral vacancies reside within the first few subsurface layers [36]. In practice, this translates into a larger heterogeneity of electron density and local magnetic moments across nonnequivalent Fe sites on (110) rather than on (111), which enhances site-specific adsorption energetics and facilitates redox-active binding motifs at undercoordinated ${\mathrm{Fe}}^{3+}$ centers once the surface is hydroxylated or partially reduced (i.e., when Fe-O coordination is dynamically altered by adsorbates) [36].

### 2.4. Magnetic Behavior

Magnetism is a defining feature of the impressive properties of iron oxides. It strongly couples to the structure, defects, and impacts the finite-temperature dynamics. Hematite (α-${\mathrm{Fe}}_{2}$$O_{3}$) is a canted antiferromagnet (weak ferromagnetism above the Morin transition point), while magnetite (${\mathrm{Fe}}_{3}$$O_{4}$) and maghemite (γ-${\mathrm{Fe}}_{2}$$O_{3}$) are ferrimagnets whose magnetic response is strongly influenced by surface anisotropy, vacancy/disorder, and size effects [39].

**Figure 4:**
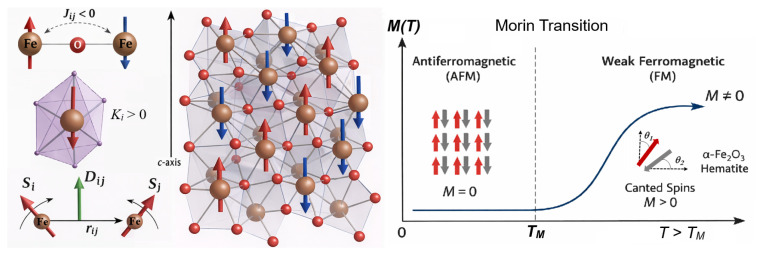
Spin interactions in hematite (α-${\mathrm{Fe}}_{2}$$O_{3}$); exchange interactions ($J_{ij}$); magnetic anisotropy ($K_{i}$); Dzyaloshinskii–Moriya interaction ($D_{ij}$). The crystal structure of hematite is shown with ${\mathrm{Fe}}^{3+}$ ions in octahedral coordination and antiferromagnetically aligned spins exhibiting weak canting due to anisotropy.

Hematite hosts a pletora of magnetic interactions, such as the exchange integral, magnetic anisotropy, and Dzyaloshinskii–Moriya interaction in addition to relativistic effects. The interplay between these named interactions induces spin canting above the Morin transition temperature at about 260 K from a weak ferromagnetic ordering at higher temperatures to antiferromagnetic at lower ones [40]. Figure 4 illustrates spin interactions in hematite (α-${\mathrm{Fe}}_{2}$$O_{3}$) and the Morin transition.

The ferrimagnetic behavior and the relatively small bulk magnetocrystalline anisotropy make magnetite highly sensitive to surface anisotropy and finite-size effects. The A–B superexchange interaction in magnetite is strongly antiferromagnetic, forcing spins on A (${\mathrm{Fe}}^{3+}$) sites to align antiparallel to those on B (${\mathrm{Fe}}^{2+}$/${\mathrm{Fe}}^{3+}$) sites. Because the B sublattice contains both ${\mathrm{Fe}}^{2+}$ and ${\mathrm{Fe}}^{3+}$ ions while the A sublattice has only ${\mathrm{Fe}}^{3+}$, their magnetic moments do not cancel, resulting in a non-zero net magnetization (ferrimagnetism).

**Figure 5:**
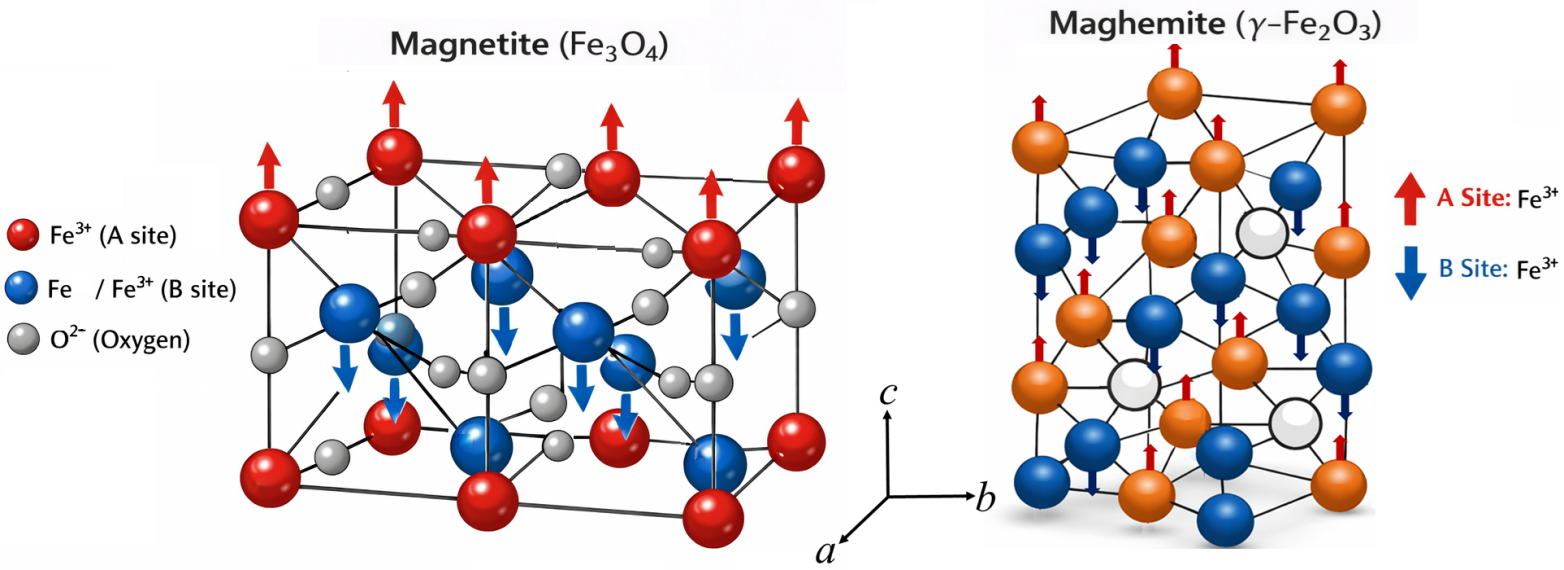
Spin interactions in magnetite (${\mathrm{Fe}}_{3}$$O_{4}$) and maghemite (γ-${\mathrm{Fe}}_{2}$$O_{3}$); blue and red arrows indicate antiparallel spin alignment on different sublattices; transparent sites corresponds cation vacancies in γ-${\mathrm{Fe}}_{2}$$O_{3}$. Crystallographic axes are indicated in the figure.

Maghemite is a vacancy-bearing spinel derivative, and vacancy/disorder can strongly influence the inherent magnetic relaxation and surface spin structure. The ${\mathrm{Fe}}^{3+}$ moments on the tetrahedral (A) site are collinearly aligned and are antiferromagnetically coupled to the octahedral (B) site by a strong A–oxygen–B superexchange that dominates the magnetic ordering. The ${\mathrm{Fe}}^{3+}$ moments on the B site have partial occupancy due to cation vacancies, which reduces the magnetization of the B site, leading to incomplete compensation and a net ferrimagnetic moment. Figure 5 shows spin interactions in magnetite (${\mathrm{Fe}}_{3}$$O_{4}$) and maghemite (γ-${\mathrm{Fe}}_{2}$$O_{3}$).

## 3. Molecular Dynamics Methodologies for Iron Oxides

MD simulations have become a pivotal tool for elucidating the atomic-scale behavior of iron oxides, bridging the gap between experimental observations and theoretical predictions. Despite the huge progress in this direction, accurate MD simulations of iron oxides remains nontrivial due to their mixed ionic–covalent bonding character, strong electronic correlations, and complex magnetic ordering. Consequently, the choice of suitable interatomic potentials or electronic-structure models is crucial. In this context, it is important to distinguish between the energetic models used to describe atomic interactions, such as DFT or interatomic potentials, and the simulation methodologies employed to explore the corresponding potential energy surface, such as MD. While the former defines the energy landscape of the system, the latter provides a framework for sampling configurations and studying time evolution. Various atomistic simulation approaches—including classical, reactive, ab initio, atomisitic spin-lattice, and machine learning-based approaches—offer complementary capabilities depending on the targeted specific properties, such as system size and surface characteristics. For a state-of-the-art review on atomistic modeling, the interested reader may consult Refs. [41,42] and the references therein. Within the scope of the MD methodologies discussed in this review, “interface” primarily denotes iron oxide surfaces in contact with gas-phase or liquid-phase environments, including aqueous and adsorbate-containing systems, while solid/solid interfaces are out of scope. Here, we will give an account of force fields commonly used in studies on the iron oxides under consideration.

### 3.1. Classical Force Fields

#### 3.1.1. Buckingham and Coulomb Potentials

Rigid-ion force fields that combine long-range electrostatics with short-range Buckingham repulsion/dispersion are standard classical force field models used in MD simulations for iron oxides, particularly when the target observables are the structural relaxation, lattice dynamics, diffusion, and large-scale surface/interface phenomena. The Lewis–Catlow framework [2] established a widely used approach for deriving Buckingham–Coulomb parameter sets for ionic oxides (including Fe-O), typically fitted to reproduce experimental lattice constants, elastic constants, and vibrational properties. For iron oxides, the central practical limitation is that fixed-charge models do not explicitly describe changes in Fe oxidation state, polaron formation, or magnetic exchange; nevertheless, they often provide robust structural fidelity for large cells and long time scales, enabling statistically converging interfacial dynamics.

MD simulations in conjunction with Buckingham-type interatomic potentials have been used to study the thermally activated mobility of atoms and the structural evolution of ${\mathrm{Fe}}_{2}$$O_{3}$ nanostructures. For example, Liu et al. [43] employed the Buckingham potential to simulate high-temperature aggregation mechanisms of ${\mathrm{Fe}}_{2}$$O_{3}$ nanoparticles, explicitly discussing the appropriateness of the Buckingham FF for ionic oxides.

Atomistic simulations of magnetite (${\mathrm{Fe}}_{3}$$O_{4}$) surfaces using classical force fields have been widely applied to study surface relaxation, adsorption energetics, and interfacial structure. Early empirical-potential studies examined water and small-molecule adsorption, highlighting the relative stability of the (111) termination and its surface-specific relaxation upon hydration [44]. While fixed-charge classical MD remains effective for long-time scale sampling of adsorption and structural dynamics on magnetite (111) compared to more open surfaces such as (110), redox-driven charge transfer and polaronic effects require methods beyond rigid-ion models.

Maghemite (γ-${\mathrm{Fe}}_{2}$$O_{3}$) is a vacancy-bearing spinel in which cation vacancy ordering governs thermodynamic stability and can affect surface stoichiometry. Classical MD has recently examined its thermally driven structural and dynamical behavior, including vacancy-containing nanoparticle models [45]. While bulk vacancy thermodynamics can be treated with classical MD, surface-specific vacancy ordering on (111) is more often addressed using electronic-structure methods or inferred from nanoparticle morphologies, highlighting an opportunity for slab-based MD under realistic hydration and adsorption conditions.

#### 3.1.2. CLAYFF

CLAYFF is a nonreactive, flexible force field designed for hydrated mineral systems, combining fixed-charge electrostatics with Lennard–Jones (12-6) short-range interactions [46]. Because CLAYFF is parameterized to be compatible with common water models and hydroxylated oxide terminations, it has become a practical choice for long-timescale MD sampling of iron-oxide/aqueous interfaces where interfacial structure (rather than redox chemistry) is the primary target.

A key CLAYFF-based application to hematite is the MD study of water structure at multiple hematite–water interfaces carried out by Kerisit [47], which explicitly considered several terminations and compared computed interfacial water structure against surface X-ray scattering models. Importantly, for surface-sensitive discussions, this work included the hematite (110) interface and highlighted how termination/protonation and surface functional groups control water layering, orientations, and hydrogen bond configurations at the interface [47]. Such simulations provide statistically converging interfacial structural descriptors (density profiles, orientational distributions, residence times) that are difficult to extract experimentally and are often used to benchmark newer potential refinements.

For magnetite, Konuk et al. [48] developed an empirical force field description for the (001) and (111) surfaces in water by leveraging CLAYFF as a baseline and introducing surface-specific charge/parameter refinements motivated by electronic-structure data, enabling MD sampling of charge redistribution effects at hydrated magnetite interfaces. In a more application-oriented classical MD, Ivanova et al. [19] employed a ClayFF-type model (with Fe-specific modification) to investigate temperature-dependent adsorption of ammonia at a magnetite {111} surface in aqueous solution. Together, these studies illustrate the typical CLAYFF usage pattern for magnetite: (i) a transferable baseline for mineral–water interactions, and (ii) targeted refinements to better represent surface termination and interfacial electrostatics. More recently, Siani et al. [49] proposed a bottom-up protocol that integrates first-principles data with classical force fields to construct and simulate pristine and PEG-coated magnetite nanoparticles in physiological conditions, resolving coating morphology, polymer ordering, and hydrogen-bonding patterns that control biofunctional performance.

A recent work by Carman et al. [50] has also used CLAYFF (including hematite-specific modifications) in nonequilibrium MD to quantify transport-related properties at iron-oxide interfaces. They compared multiple solid interaction models—including a Kerisit-modified CLAYFF variant and INTERFACE-FF—to compute interfacial thermal resistance across hematite–hydrocarbon interfaces, highlighting how force field choice impacts interfacial structure and transport observables.

Direct CLAYFF-based MD studies [51] explicitly targeting vacancy-ordered γ-${\mathrm{Fe}}_{2}$$O_{3}$ (maghemite) surface slabs (e.g., (111)) remain comparatively scarce; in practice, CLAYFF is most commonly used for hydrated interface sampling when a suitable maghemite parameterization is available or can be transferred/adjusted from related Fe-O-H descriptions.

#### 3.1.3. INTERFACE-FF

INTERFACE-FF is a thermodynamically consistent, nonreactive force field designed to model inorganic–organic and inorganic–aqueous interfaces with improved transferability compared to preceding Lennard–Jones–Coulomb models [52]. Like CLAYFF, it employs fixed partial charges and Lennard–Jones (12-6) interactions, but the model parameters are derived or validated against thermodynamic reference data, including surface and interfacial energies, hydration free energies, and related experimental or first-principles benchmarks across oxide materials.

A key recent development for bio/nano simulations is the extension of INTERFACE-FF surface models to α-${\mathrm{Fe}}_{2}$$O_{3}$ with validated surface and interfacial properties and direct compatibility with mainstream biomolecular force fields (e.g., AMBER/CHARMM), enabling large-scale simulations of oxide-polymer/protein environments [53]. In application-driven studies of iron-oxide nanoparticles (maghemite) in biological fluids, Mekseriwattana et al. [54] combined proteomics with atomistic MD to rationalize how surface hydrophilicity and ligand chemistry of riboflavin-coated superparamagnetic iron oxide nanoparticles (SPIONs; typically magnetite/maghemite based) modulate binding to a target protein (riboflavin carrier protein) and influence serum-derived protein corona formation.

The principal advantage of INTERFACE-FF lies in its thermodynamic consistency across inorganic and organic phases, enabling reliable predictions of adsorption free energies and interfacial morphology. However, as with other fixed-charge models, it does not explicitly treat redox chemistry, electronic polarization, or magnetic exchange interactions, which are central to strongly correlated iron oxides.

#### 3.1.4. Core–Shell (Polarizable) Models

To incorporate ionic polarizability of iron oxides, core–shell models represent polarizable ions (typically $O^{2-}$) by a massive core and a massless shell connected by a harmonic spring [55]. For iron oxides, shell-model parameterizations based on the formalism of Lewis and Catlow [2] have been used to improve the description of dielectric response, phonon dispersion, and defect energetics in hematite and magnetite. In maghemite, where cation vacancies induce local charge redistribution, explicit polarization can improve structural stability upon rigid-ion models. However, the increased computational cost limits large-scale surface simulations compared to non-polarizable Buckingham-type potentials.

### 3.2. Reactive Force Fields

Reactive FFs, most prominently ReaxFF, enable bonds forming and breaking in conjunction with dynamic evolution of atomic charges, providing access to chemistry processes (hydroxylation, oxidation/reduction and phase transformation) [3,56,57,58]. To describe the Fe–O–H chemistry, dedicated parametrizations protocols are required to maintain transferability across oxidation states, coordination environments, and interfacial water [15,56].

ReaxFF has been used to model adsorption and thermal stability of organic binders on hematite surfaces, capturing temperature-driven bond rearrangements and decomposition pathways that are inaccessible to nonreactive force fields [59]. A notable surface-specific development relevant to low-index facets is the force-matching-based refitting workflow demonstrated for a hematite (110) slab system, which explicitly targets the stability and reactivity of a (110) surface model under interaction with an oxide cluster [60]. This provides a practical route for improving ReaxFF robustness on open, undercoordinated terminations (such as (110)), where unphysical surface instabilities can otherwise occur. Direct ReaxFF studies that explicitly contrast hematite (110) and (111) slabs in reactive environments remain comparatively limited; consequently, many reviews discuss (110) vs. (111) reactivity trends by combining (i) ReaxFF-enabled MD simulations on specific slabs (commonly (110) or (001)) with (ii) electronic-structure benchmarks for close-packed terminations, to rationalize how coordination and protonation modulate water dissociation barriers and hydroxyl stability.

Magnetite’s mixed-valence character makes oxidation and reduction pathways central to its modeling. ReaxFF studies of iron oxidation/corrosion environments provide mechanistic insight into oxygen uptake, oxide-film growth, and defect evolution that are directly relevant to magnetite-forming conditions. For example, ReaxFF MD has been applied to study wet oxidation on Fe-Cr alloy surfaces (water adsorption/dissociation and subsequent oxide growth) [61], as well as oxidation of Fe in supercritical ${\mathrm{CO}}_{2}$/$O_{2}$ mixtures [62]. In chloride-containing electrolytes, ReaxFF simulations have been used to resolve early-stage depassivation and oxide-film formation processes [63]. While these corrosion studies are typically performed on metallic Fe surfaces, the emergent oxide products and film evolution connect directly to magnetite-bearing corrosion scales.

Maghemite often appears as an oxidation product/intermediate of magnetite and can be stabilized by vacancy ordering in the spinel framework. ReaxFF MD has been used to model the generation and growth of iron oxide nanoparticles from representative precursors, providing a chemically reactive route to Fe$O_{x}$ nanoparticle formation at atomistic resolution [64]. A recent combined experimental-simulation study employed ReaxFF MD to interrogate hematite-to-magnetite reduction pathways that proceed either directly or via maghemite as an intermediate, linking atomistic transformation mechanisms to microstructural strain signatures [65]. Such work illustrates the capability of ReaxFF for describing transformation sequences among hematite, magnetite, and maghemite under reactive environment.

Beyond widely used approaches such as ReaxFF, alternative variable-charge many-body potentials have also been developed. In particular, charge-optimized many-body (COMB) potentials, especially within the COMB3 formalism, explicitly account for dynamically varying atomic charges and incorporate many-body interactions, making them suitable for systems with mixed ionic–covalent bonding [66,67]. These approaches have been successfully applied to a range of oxide materials. However, their application to iron oxides remains limited, and no broadly validated parameter sets are currently available for major phases such as ${\mathrm{Fe}}_{2}$$O_{3}$ or ${\mathrm{Fe}}_{3}$$O_{4}$. This likely reflects the complexity associated with describing the strongly correlated electronic structure and multiple oxidation states of iron.

In reactive simulations, surface orientation controls undercoordination, hydroxyl stability, and access to dissociation pathways. Open terminations (often (110)-type) tend to show higher densities of reactive sites and stronger surface reconstruction tendencies, demanding careful validation of ReaxFF parameter stability on slabs [60]. Close-packed (111)-type terminations are typically less prone to large-amplitude reconstruction but may require accurate treatment of protonation states and electrostatics to capture realistic hydroxylation and water-mediated chemistry. In practice, explicit ReaxFF datasets for iron-oxide (111) slabs remain less common than for more open terminations and metallic Fe surfaces, motivating continued development of facet-specific analysis and validation protocols.

### 3.3. Ab Initio Molecular Dynamics for Iron Oxides

DFT-based ab initio methods compute interatomic forces “on the fly” from the electronic structure, enabling chemically faithful finite-temperature sampling of iron-oxide surfaces and interfaces where bond rearrangements, proton transfer, and electronic localization effects play a central role. Most modern studies adopt Born–Oppenheimer MD, while Car-Parrinello MD provides an alternative extended-Lagrangian framework [68]. For ionic and strongly correlated iron oxides, spin polarization and Hubbard-corrected DFT (DFT+*U*) are typically required to obtain reasonable local moments and Fe-O bonding, especially at hydrated interfaces. In the examples hereafter, conclusions regarding hydrogen bonding, proton transfer, water dissociation, and interfacial restructuring are based on direct analysis of MD trajectories, whereas statements regarding electronic localization, charge redistribution, and bonding character should be understood as DFT-based electronic-structure interpretations extracted from those simulations.

AIMD has been used extensively to unravel the structure, protonation, and reactivity of hematite–water interfaces beyond static DFT, including interfacial acid–base equilibria and electric field-driven chemistry. Futera et al. [16] used nonequilibrium ab initio MD to investigate dynamical properties of adsorbed water molecules on the hematite (001) surface, including hydrogen bonding and dissociation dynamics. Gittus et al. [69] computed acidity constants of the hematite–liquid water interface from AIMD, establishing a route to connect atomistic sampling to surface protonation thermodynamics. Ulman et al. [70] applied first-principles MD to probe the electrochemical double layer at the hematite/water interface, highlighting how interfacial water organization and surface charging respond to electrochemical conditions. Electric-field and nonequilibrium AIMD studies have further shown that external fields can promote water dissociation at hematite interfaces. Futera and English [16] investigated water breakup at hematite/water interfaces under applied electric fields using nonequilibrium AIMD, while Ajide et al. [71] performed nonequilibrium AIMD simulations of water splitting at hematite/water interfaces in an external electric field, resolving dynamical pathways for field-assisted dissociation at room temperature. These AIMD results provide critical benchmarks for reactive force fields and for machine learning interatomic potentials trained on DFT data.

AIMD for magnetite interfaces is more challenging than for many oxides because mixed valence, electron hopping/polaron formation, and magnetic order complicate convergence and MD simulations. Nevertheless, recent first-principles MD has been used to directly address reactive interfacial phenomena. Wang et al. [72] employed first-principles MD to study high-temperature water interaction with ${\mathrm{Fe}}_{3}$$O_{4}$(111), targeting interfacial reaction and charge-transfer behavior under hydrothermal conditions. Here, the reaction pathways and water dynamics are supported by first-principles MD, while the discussion of charge-transfer behavior relies on the underlying DFT-based electronic-structure description. Chandy et al. [73] combined AIMD and free-energy calculations to investigate reaction mechanisms at water–nitrogen–magnetite interfaces relevant to ambient-condition nitrogen chemistry.

AIMD has also been coupled with experiment to interrogate redox-driven dynamical processes: Wu et al. [74] combined ^57^Fe Mössbauer spectroscopy with first-principles MD to elucidate Fe atom exchange at the magnetite–water interface under redox driving forces. Finally, ligand-induced restructuring and facet-dependent chemistry can be captured by AIMD-enabled surface sampling; for example, Ortiz-Garcia et al. [75] report acid-induced restructuring of ${\mathrm{Fe}}_{3}$$O_{4}$(001), explicitly relying on AIMD to manage the complex electronic/magnetic landscape of magnetite surfaces.

Compared with hematite and magnetite, AIMD studies explicitly targeting maghemite are less common, in part because vacancy ordering and multiple metastable cation/vacancy configurations expand the configurational space. Still, DFT-based MD has been used to probe interfacial electronic structure and charge redistribution in contact with water. Ntallis et al. [76] studied charge distribution at the water/γ-${\mathrm{Fe}}_{2}$$O_{3}$ interface using DFT MD for both thin-film and nanoparticle models, providing direct insight into water-induced charge rearrangement in vacancy-bearing γ-${\mathrm{Fe}}_{2}$$O_{3}$ motifs. In this case, the interfacial configurations are sampled by MD, while the charge distribution and electronic response are obtained from the DFT electronic structure along the trajectory. This type of AIMD evidence is particularly valuable for assessing when fixed-charge classical models (or even charge-equilibration approaches) may fail to reproduce interfacial polarization and defect-mediated electronic response in maghemite.

AIMD is most defensible for (i) interfacial proton transfer and hydroxylation chemistry, (ii) field/electrochemical effects, (iii) redox-coupled surface reactions, and (iv) generating reference data for machine learning interatomic potentials. The principal limitations are the accessible short timescales and the sensitivity of iron oxides to the chosen DFT functional, *U* value, and magnetic configuration. Accordingly, conclusions drawn from AIMD should be distinguished from static DFT interpretations—the former directly support finite-temperature structural and dynamical behavior, whereas the latter primarily support electronic, magnetic, and bonding analyses. For maghemite in particular, systematic AIMD comparisons across vacancy-ordering patterns and low-index facets (e.g., (111)) remain a clear opportunity for future work.

### 3.4. Machine Learning Interatomic Potentials for Iron Oxides

Machine learning interatomic potentials (MLIPs) aim to reproduce ab initio potential-energy surfaces at a fraction of the computational cost, enabling MD simulations of iron oxides at length and time scales inaccessible to AIMD while retaining near-DFT fidelity [77,78,79,80]. Modern MLIPs typically learn atomic energies from local structural descriptors (e.g., high-dimensional neural network potentials, Gaussian approximation potentials, or message-passing graph networks) and are trained to DFT (often DFT+*U* for Fe oxides) energies and forces. A recently developed [81] machine learning potential built upon the Atomic Cluster Expansion [82] was shown to reproduce the thermodynamics properties of Fe-O systems spanning the whole range of oxygen content and accounting for magnetic degrees of freedom.

A central application domain for hematite is the oxide–water interface, where accurate treatment of interfacial hydrogen bonding, hydroxylation, and surface protonation requires electronic-structure quality but benefits from long trajectories and large interfacial cells. Shiranirad and English [83] developed a machine-learned potential for MD of hematite–water interfaces using a pool-based active-learning strategy to address incomplete force labeling in the reference dataset. Their MLIP enables extended MD sampling of interfacial structure while targeting DFT-level accuracy, illustrating how active learning can reduce the cost of generating representative training configurations for complex hydrated oxide surfaces.

For magnetite, the combination of mixed valence, strong correlation, and complex surface reconstructions makes long-timescale interfacial sampling particularly challenging with AIMD. Romano et al. [84] developed a Behler–Parrinello neural network potential for the magnetite/water system and performed extensive MD simulations of the magnetite (001)/water interface over a wide range of water coverages. The neural network potential-based MD reproduced pronounced interfacial water layering, provided quantitative diffusion anisotropy at low coverage, and delivered mechanistic insight into water dissociation events on the reconstructed surface. This work exemplifies how MLIPs can bridge AIMD and classical MD to access facet-specific interfacial dynamics on magnetite.

Compared with hematite and magnetite, MLIP-driven MD studies explicitly targeting vacancy-ordered maghemite slabs (e.g., (111)) remain relatively scarce, largely because vacancy ordering substantially expands the configurational space that must be represented in training data. Nevertheless, recent MLIP development has begun to address multi-phase Fe-O chemistry in a unified way. Torres et al. [85] reported a machine learning interatomic potential trained on DFT+*U* data for hematite with demonstrated transferability to other bulk iron oxides, providing an important foundation for future maghemite-focused MD (including vacancy-bearing spinel configurations). Extending such models to facet-resolved (111) and (110) surface slabs under hydration/adsorption conditions is a clear direction for enabling predictive, large-scale MD of maghemite interfaces.

Because iron oxides are sensitive to functional choice, Hubbard *U*, and magnetic configuration, best practice is to (i) curate training sets spanning relevant oxidation states, coordinations, and surface terminations; (ii) validate against ab initio benchmarks for surface energies, adsorption geometries, and interfacial structural observables; and (iii) use active learning/on-the-fly selection to reduce extrapolation risk in production MD [86]. In practice, MLIPs are most impactful when used to generate statistically converged interfacial properties (structure, diffusion, rare events with enhanced sampling) while retaining a tight coupling to electronic-structure reference calculations for calibration and uncertainty control.

### 3.5. Comparison of Atomistic Simulation Frameworks

Atomistic modeling of iron oxides spans a hierarchy of simulation methodologies that differ in physical fidelity, computational cost, and accessible time and length scales. A summary is provided in Table 2.

Classical force fields provide computationally efficient frameworks for MD simulations of bulk and surface behavior of hematite, magnetite, and maghemite. Buckingham-based and CLAYFF-type models are most widely used for (110) and (111) surface studies, while polarizable approaches extend applicability to dielectric and mechanical phenomena. AIMD provides first-principles accuracy but is limited in system size and timescale. Spin-lattice dynamics provide a framework for coupling atomic motion with magnetic degrees of freedom and is therefore useful for studying magnetoelastic effects, spin–phonon coupling, and temperature-dependent magnetic behavior in iron oxides. Machine learning interatomic potentials bridge AIMD accuracy and classical scalability when trained on representative DFT datasets. The appropriate methodology depends on whether the target properties involve surface hydroxylation, defect ordering, mechanical response, or interfacial adsorption.

## 4. Adsorption of Small Molecules on Iron Oxide Surfaces

MD simulations provide useful atomistic insights into adsorption geometries, hydrogen-bonding networks, residence times, and facet-dependent reactivity of iron oxide surfaces. Because adsorption behavior depends strongly on the simulation environment, the results discussed below are interpreted with respect to whether the models include explicit aqueous solvent, vacuum slab conditions, and specific surface hydroxylation or protonation states. Across hematite (α-${\mathrm{Fe}}_{2}$$O_{3}$), magnetite (${\mathrm{Fe}}_{3}$$O_{4}$), and maghemite (γ-${\mathrm{Fe}}_{2}$$O_{3}$), the contrast between compact (111) terminations and more open (110) surfaces plays a central role in modulating adsorption strength, interfacial water structure, and the availability of coordinatively unsaturated Fe sites. This emphasis on (111) and (110) surfaces is intended to provide a comparative framework between relatively compact and more open/reactive terminations across the iron oxide phases considered here. However, the (001) surface is also highly relevant, particularly for magnetite, where reconstruction, water adsorption, and reactive surface processes have been extensively studied. Therefore, ${\mathrm{Fe}}_{3}$$O_{4}$(001) is discussed where appropriate, while the (111)/(110) comparison is used as the main organizing framework for identifying facet-dependent trends.

### 4.1. Water Adsorption

Classical MD studies of hematite–water interfaces have demonstrated pronounced surface dependence in hydration structure. Kerisit [47] reported systematic simulations of water at several hematite facets, including (110), showing that surface topology strongly shapes interfacial density oscillations, hydrogen bond networks, and orientational ordering of the first hydration layer. The more open (110) surface exposes undercoordinated Fe sites that promote heterogeneous hydrogen bonding and stronger interfacial structuring compared to more compact terminations.

Beyond fixed-charge models, hybrid-DFT MD revealed detailed hydrogen bond statistics and surface–water electronic coupling at the hematite/liquid interface [89]. Field-driven nonequilibrium AIMD further demonstrated that electric fields can promote water dissociation and proton transfer at hematite-water interfaces, directly linking surface electronic structure to dynamic interfacial chemistry [16,71]. Complementary field-dependent dynamics has also been analyzed in terms of enhanced mobility of adsorbed interfacial water under applied fields [90]. Recent machine learning potentials trained on hybrid DFT enable nanosecond-scale simulations of hematite/water interfaces while retaining near-DFT fidelity, allowing convergence of water residence and diffusion statistics inaccessible to short AIMD runs [91].

For magnetite, MD studies have clarified the structure of hydrated surfaces and the importance of accurate oxide–water cross-interactions. Classical simulations of ${\mathrm{Fe}}_{3}$$O_{4}$/water interfaces established interfacial layering and hydroxyl stabilization patterns under aqueous conditions [92]. More recently, multiscale MD of ${\mathrm{Fe}}_{3}$$O_{4}$(001)/water combined electronic-structure validation with classical sampling to characterize coverage-dependent adsorption motifs [17].

Although many detailed MD investigations focus on reconstructed (001) surfaces, the structural dissimilarity between close-packed (111) and more open (110) facets suggests comparable trends: compact (111) terminations tend to stabilize ordered water adsorption layers, whereas (110) surfaces are expected to exhibit greater adsorption heterogeneity and potentially enhanced dissociation due to the lower Fe coordination. Improved Fe-$O_{\mathrm{water}}$ cross-interaction parameterizations now enable transferable classical MD of hydrated magnetite nanoparticles and surfaces [93], while neural network potentials extend simulations toward DFT-level accuracy [84].

Water adsorption on maghemite is complicated by intrinsic cation vacancies within the defect spinel lattice. DFT-based MD has shown that interfacial water can induce significant electronic polarization at the water/γ-${\mathrm{Fe}}_{2}$$O_{3}$ interface [76]. Since vacancy ordering modifies local Fe coordination, hydration structure on (111) and (110) facets is expected to depend sensitively on vacancy distribution. However, systematic facet-resolved classical MD for vacancy-ordered γ-${\mathrm{Fe}}_{2}$$O_{3}$ slabs remains limited, representing a key opportunity for future research.

### 4.2. Nitrogen-Containing Molecules

Nitrogen-containing adsorbates (${\mathrm{NH}}_{3}$, amines, diamines, and related N-bearing organics) are pertinent to corrosion-control chemistry, surface functionalization, and catalytic nitrogen conversion. Classical MD has increasingly been applied to probe adsorption and interfacial behavior of small nitrogen-containing molecules on iron-oxide surfaces, extending beyond simple ammonia to multifunctional amines under realistic aqueous conditions.

A complementary molecular mechanics/MD strategy for organic/oxide interfaces was proposed by Rey et al. [94], who introduced an additional attractive Gaussian term to capture the adsorption of oxygenated and amine molecules on hematite surfaces, validating against reference data and enabling more realistic adsorption structures within classical MD workflows. Such approaches are useful when the conventional Lennard–Jones potential along with electrostatics underestimate specific Fe-N binding or fail to reproduce experimentally consistent adsorption motifs.

**Figure 6:**
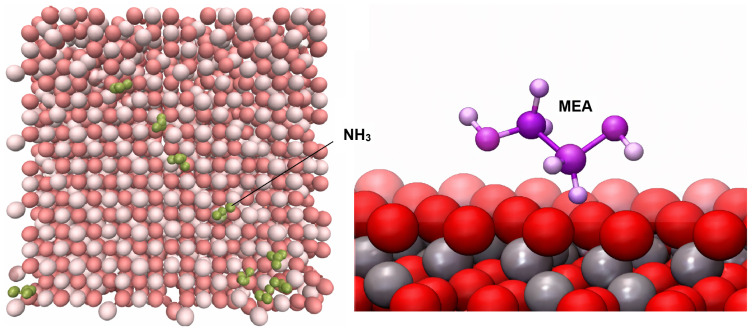
Ammonia, ${\mathrm{NH}}_{3}$ (in dark green) adsorption on the surface is shown in top view; Monoethanolamine, MEA (in purple) adsorption on magnetite is presented in side view, illustrating its binding geometry and interaction with undercoordinated surface atoms.

A recent comprehensive MD study examined ammonia adsorption on magnetite’s (${\mathrm{Fe}}_{3}$$O_{4}$) {111} surface in an aqueous phase over a broad temperature range (293–473 K) using a ClayFF model with modified Fe parameters. Trajectory analysis (up to 200 ns) revealed that ammonia gradually approaches and binds near the surface oxygen lattice, with the extent and kinetics of approach influenced by temperature. Radial distribution functions and root-mean-square deviation profiles provided clear signatures of ammonia proximity to ${\mathrm{Fe}}_{3}$$O_{4}$(111), capturing facet-specific dynamics and surface–adsorbate structuring [19]. Extended MD work on monoethanolamine and its protonated form on magnetite (111) also used ClayFF to quantify the adsorption thermodynamics and the underlying mechanism. These simulations demonstrated that (i) monoethanolamine adsorption is driven by electrostatic interactions between the ${\mathrm{NH}}_{2}$ moiety and surface oxygen/Fe centers, (ii) adsorption rates increase with temperature, and (iii) charged ethanol–ammonium cations approach the surface faster than neutral monoethanolamine. Adsorption energies calculated as a function of temperature highlighted distinct aspects of molecular approach and surface involvement [20]. Nitrogen-containing organic compounds have also been investigated in the context of adsorption and segregation of amine crosslinkers at iron oxide interfaces. On Figure 6, ammonia and monoethanolamine adsorption on magnetite are shown. Harris et al. [95] performed atomistic MD of epoxy/amine mixtures on hematite and magnetite surfaces (CLAYFF-type surface description), quantifying preferential adsorption of amine nitrogen at the solid interface and demonstrating how surface identity alters interfacial composition and binding site distributions.

Although most nitrogen-containing MD adsorbate studies have targeted magnetite (111), the higher density of undercoordinated Fe on open (110) surfaces suggests stronger Lewis-acid interactions for nitrogen groups, potentially enhancing adsorption strength and facilitating hydrogen bond network formation with water. However, systematic MD comparisons for (110) facets across hematite, magnetite, and maghemite are limited. A robust comparison will require validated surface models and possibly reactive or machine learning-trained potentials to capture proton-coupled pathways and charge redistribution accompanying nitrogen adsorption, especially under aqueous/thermal conditions.

### 4.3. Organic Molecules

Organic adsorption on iron oxides is relevant to nanoparticle functionalization (ligands/surfactants), environmental uptake (aromatics/dyes), and surface modification for catalysis and separations. In MD, binding typically emerges from the interplay of (i) specific coordination of polar headgroups (e.g., carboxylate/phosphonate/hydroxyl) to surface Fe sites, (ii) hydrogen bonding to surface oxygen/hydroxyl groups, and (iii) hydrophobic packing and lateral ordering at higher coverages.

#### 4.3.1. Carboxylic Acids and Carboxylates on Magnetite

A thorough MD analysis with facet calculation is provided by the adsorption of oleic acid on magnetite: Creutzburg et al. [96] combined surface experiments with MD simulations to determine adsorption geometries and coverage-dependent ordering on magnetite (111) (and (001)), showing dissociative adsorption at low coverages and stronger lateral association/upright orientation at higher coverages, with distinct molecular layer structure on (111). This work illustrates how compact magnetite facets can still host strong chemisorption through carboxylate anchoring while exhibiting facet-dependent packing and hydrogen bond patterns within the organic adlayer.

Shorter carboxylates provide additional prototypical MD benchmarks: heterogeneous adsorption and local ordering of formate on magnetite surfaces have been probed by simulations related to the surface structure and termination, highlighting that lateral ordering and site heterogeneity can coexist even for small organics [97]. Together, these studies support the common workflow of using small carboxylates as calibration systems prior to simulating larger ligand shells.

#### 4.3.2. Phenols and Substituted Aromatics

For aqueous-phase aromatics, classical MD has been used to quantify preferred orientations and adsorption energetics on iron oxide substrates. A MD study of phenol, *p*-chlorophenol, and *p*-nitrophenol on magnetite showed that adsorption depends on the interplay between surface affinity and hydration stabilization, with water-bridged hydrogen bonding contributing to interfacial anchoring and orientational preferences [98]. Complementary MD analysis for phenol on hematite surfaces indicates that solvent may enhance adsorption by promoting closer approach and stabilizing surface-bound configurations through water-mediated interactions [99]. While such studies typically focus on a single termination, they motivate systematic facet-resolved comparisons (111 vs. 110) in future studies, since the open (110) geometry should increase adsorption-site diversity and strengthen specific binding patterns for oxygenated aromatics.

#### 4.3.3. Liquid-Phase Organics and Large-Scale Adsorption

Reactive MD has been used to assess how organic polarity couples to surface relaxation and polarization on hematite. Chia et al. [100] employed ReaxFF-based MD to compare adsorption of organic liquids of different polarity on hematite (α-${\mathrm{Fe}}_{2}$$O_{3}$), demonstrating that flexible/polarizable surface descriptions capture adsorption-induced structural response that is absent in rigid-surface treatments. This highlights a key methodological point for organic adsorption on hematite: interfacial polarization and surface relaxation can be non-negligible, particularly for strongly polar organics. For maghemite (γ-${\mathrm{Fe}}_{2}$$O_{3}$), organic adsorption MD is often reported for nanoparticle/composite models rather than ideal (111)/(110) slabs. Ouachtak et al. [101] combined experiment with MD simulations to investigate adsorption of Rhodamine B on a maghemite-based magnetic composite, using MD to rationalize adsorption configurations and interaction modes at the surface of the magnetic phase. While not facet-resolved, these simulations provide practical evidence for how organic dyes interact with vacancy-bearing spinel-derived magnetic materials at realistic length scales.

Beyond adsorption processes relevant to aqueous, catalytic, and environmental interfaces, atomistic modeling of iron oxide surfaces is also relevant to metallurgical reduction and metal extraction. These applications involve interfacial reactions between iron oxides and reactive species such as $H_{2}$, CO, CO/$H_{2}$ mixtures, hydrocarbons, and carbon-based reductants. Reactive MD simulations have begun to address such processes at the atomic scale. For example, ReaxFF MD has been applied to hydrogen interaction with FeO and ${\mathrm{Fe}}_{2}$$O_{3}$, providing atomistic insight into $H_{2}$ adsorption, dissociation, and the initial stages of oxide reduction [58]. More recently, combined experimental and ReaxFF MD studies have examined ${\mathrm{Fe}}_{2}$$O_{3}$ reduction by $H_{2}$/CO mixed gases, showing how reactive simulations can clarify oxygen removal, charge redistribution, and the role of CO in promoting reduction pathways [102]. ReaxFF MD has also been used together with experimental observations and DFT calculations to analyze hematite-to-magnetite reduction under molecular hydrogen, linking atomistic transformation mechanisms to microstructural evolution [65]. These studies demonstrate that the methodological issues discussed in this review, including charge transfer, redox chemistry, bond breaking and formation, defect evolution, and surface reconstruction, are directly relevant to iron oxide reduction and extraction processes. However, direct MD studies of iron oxide reduction by solid carbonaceous reductants remain comparatively limited; therefore, this area represents an important opportunity for future reactive force field and machine learning potential developments.

### 4.4. Comparative Overview of Facet-Dependent Adsorption

Table 3 summarizes representative MD findings for adsorption of water, nitrogen-containing molecules, and selected organic compounds on hematite, magnetite, and maghemite surfaces, with emphasis on the contrast between compact (111) and more open (110) facets. Across the systems considered, adsorption behavior is governed by surface coordination, hydroxylation state, and electrostatic representation within the chosen force field or electronic-structure framework. When direct MD data are available, the table reports trajectory-based observations, such as interfacial layering, residence times, orientational ordering, and adsorption stability. Otherwise, facet-dependent trends are discussed as interpretations supported by static DFT calculations, electronic-structure arguments, or structural considerations. Compact (111) terminations often stabilize ordered interfacial layers and well-defined adsorption geometries, whereas open (110) surfaces expose a higher density of undercoordinated Fe sites that can enhance adsorption heterogeneity and potentially strengthen specific adsorbate-surface interactions. The table highlights both established MD results—particularly for water and carboxylate adsorption on magnetite (111)—and areas where systematic facet-resolved simulations remain limited, notably for nitrogen-containing molecules and vacancy-ordered maghemite surfaces.

Cross-study comparisons presented in Table 2 should also be interpreted cautiously. For example, hydration structure and residence times obtained with fixed-charge models such as CLAYFF or INTERFACE-FF are not directly comparable to results from ReaxFF, AIMD, or ML-based simulations, because these approaches differ in their treatment of charge redistribution, bond breaking, and surface proton transfer. Similarly, different water models and oxide–water cross-interaction parameters can change interfacial layering, hydrogen bond lifetimes, and diffusion near the surface. Finally, nominally identical facets may correspond to different slab terminations, hydroxyl coverages, protonation states, or vacancy arrangements, especially for magnetite and maghemite. Therefore, robust facet-dependent trends should ideally be extracted from simulations performed with the same force field or electronic-structure setup, surface termination, and solvation conditions, or validated across multiple models.

The simulation environment is distinguished: the water adsorption studies, the nitrogen-containing molecule studies and the adsorption of liquid-phase organics are based on aqueous-phase simulations, whereas the systems with some organic molecules are based on vacuum slab models. Consequently, comparisons between adsorption trends should account for whether explicit solvent is included and whether the surface is modeled as clean, hydroxylated, or protonated. Across water, nitrogen-containing molecules and organic adsorbates, available MD simulations indicate that surface topology, hydroxylation state, and electrostatic representation govern adsorption behavior. Close-packed (111) surfaces tend to stabilize ordered adlayers and adsorption geometries, whereas open (110) facets introduce coordination heterogeneity that can enhance adsorption diversity and potentially promote dissociation. Compared with hematite and magnetite, however, the facet-resolved MD literature on maghemite remains underdeveloped. Direct MD studies of well-defined γ-${\mathrm{Fe}}_{2}$$O_{3}$ slab surfaces, especially systematic comparisons between (111), (110), and other low-index facets, are scarce. Consequently, current discussion of maghemite surface adsorption often relies on DFT-MD studies, nanoparticle or composite models, and qualitative expectations based on vacancy ordering and surface coordination. To the best of our knowledge, explicit MD simulations of the maghemite (110) surface has not been carried out so far. However, some of these facet-dependent conclusions should be regarded as qualitative trends when they are inferred from coordination arguments or DFT-based electronic-structure results rather than from direct MD comparisons on the same material and adsorbate. The integration of validated classical force fields, reactive potentials, AIMD benchmarks, and machine learning interatomic potentials provides a pathway toward predictive, facet-resolved adsorption modeling for hematite, magnetite, and maghemite.

## 5. Open Challenges and Perspectives

Despite major advances in MD simulations of hematite (α-${\mathrm{Fe}}_{2}$$O_{3}$), magnetite (${\mathrm{Fe}}_{3}$$O_{4}$), and maghemite (γ-${\mathrm{Fe}}_{2}$$O_{3}$), accurate modeling of iron-oxide interfaces with various chemical compounds remains limited by several interconnected challenges spanning electronic structure, magnetism, surface chemistry, and timescale accessibility.

### 5.1. Accurate Treatment of Charge Transfer

A primary limitation concerns the accurate description of charge transfer and mixed valence. Magnetite and maghemite exhibit ${\mathrm{Fe}}^{2+}$/${\mathrm{Fe}}^{3+}$ coexistence and vacancy-driven charge redistribution, while hematite induces a small-polaron formation. Classical fixed-charge force fields cannot capture these effects, and even reactive potentials struggle to reproduce quantitative redox energetics. A realistic future target is to achieve adsorption and redox reaction energies within ±0.1 eV of DFT+*U* benchmarks and surface charge distributions within 5% of electronic-structure references. As proposed practical benchmarking goals rather than established universal thresholds, future models could aim to reproduce adsorption and redox reaction energies within approximately ±0.1 eV of suitable of DFT+*U* benchmarks and surface charge distributions within about 5% of reliable reference values.Machine learning interatomic potentials trained on hybrid DFT datasets offer a promising pathway, particularly if explicitly constrained to reproduce polaron localization and defect energetics.

### 5.2. Facet-Resolved Surface Chemistry

Beyond static DFT, *dynamical* simulations corroborate this facet-sensitive picture by explicitly linking surface coordination to time-dependent charge redistribution at oxide/water interfaces. DFT-based MD has been used to analyze charge displacement and electrostatic potential variations upon hydration of γ-${\mathrm{Fe}}_{2}$$O_{3}$ films and nanoparticles, reporting that water adsorption changes the ionic state of surface Fe (increasing its effective oxidation/ionization character) while leaving the overall magnetic moments comparatively less affected under the sampled conditions [76]. Complementarily, a recent classical MD work on maghemite reveals how thermal cycling and structural disorder modify local coordination statistics and dynamical response, providing a route to bridge bulk/nanoparticle structural evolution with surface-reactivity descriptors used in interfacial models [45]. For chemically specific adsorption on (111), recent DFT studies of small molecules (including $H_{2}$O) on γ-${\mathrm{Fe}}_{2}$$O_{3}$(111) directly connect PDOS-level electron transfer with adsorption site identity: octahedral and tetrahedral surface Fe act as primary binding centers, and hydroxyl pre-coverage can substantially change adsorption strengths and charge transfer pathways [103]. Collectively, these DFT and MD results establish a consistent framework in which vacancy order and facet geometry (111) vs. (110) control the distribution of Fe 3d acceptor states and O 2p donor states, thereby governing adsorption-driven charge transfer, proton-coupled processes, and ultimately the catalytic functionality of maghemite surfaces. Understanding these facet-dependent structural and electronic differences at the molecular level is essential for tuning hematite’s performance in catalysis, sensing, and energy conversion applications.

Facet-resolved surface chemistry presents a second major challenge. Although water adsorption on magnetite (111) and hematite (110) has been extensively modeled, systematic comparisons between compact (111) and open (110) surfaces across all three oxides remain incomplete. Open (110) facets exhibit a higher density of undercoordinated Fe sites, potentially altering adsorption free energies by several tenths eV and modifying dissociation pathways. For maghemite, vacancy ordering further complicates slab construction and surface stoichiometry. Future MD studies should target statistically converged adsorption free energies with clearly reported uncertainties; as a proposed practical benchmarking goal, uncertainties on the order of 1 kJ ${\mathrm{mol}}^{-1}$ would provide a stringent target for comparing facet-dependent adsorption trends, computed via enhanced sampling methods over at least tens of nanoseconds for classical or ML-based simulations.

### 5.3. Magnetic Degrees of Freedom and Spin-Lattice Coupling

Magnetic degrees of freedom introduce an additional level of complexity. Spin canting, Dzyaloshinskii–Moriya interaction, and surface anisotropy govern phenomena such as the Morin transition and nanoparticle superparamagnetism. However, most adsorption-focused MD simulations neglect explicit spin dynamics. Quantitative coupling between surface chemistry and magnetic anisotropy remains largely unexplored. As a proposed practical benchmarking goals future spin-aware models could aim to reproduce experimental Morin transition temperatures within approximately ±10 K and surface anisotropy constants within about 10% of suitable experimental or electronic-structure reference values while also capturing adsorption-induced modifications of exchange parameters.

Classical MD with reactive force fields, such as ReaxFF, can capture charge redistribution and polarization effects associated with surface interactions, adsorption, and defect formation of Hematite [56]. These simulations show that the highest occupied states are predominantly oxygen 2p in character, while the lowest unoccupied states derive mainly from Fe 3d orbitals, consistent with the semiconducting behavior of hematite. Spin-polarized DFT calculations further confirm the antiferromagnetic ordering and the crystal-field splitting of Fe 3d states within the octahedral environment [26]. An ab initio-parameterized spin model combined with atomistic spin dynamics has been used [104] to explore the hematite phase behavior and quantify the balance of exchange, dipolar terms, and anisotropies required to adequately reproduce the experimental outcome. Such studies provide a route to connect DFT-derived tensorial exchange parameters to finite-temperature magnetic phase stability and are increasingly used to rationalize surface and nanoscale magnetic behavior via modified anisotropy and broken-symmetry environments.

Atomistic spin-model simulations of magnetite have demonstrated how surface anisotropy can induce pronounced hysteresis-loop shifts and modify coercivity and remanence as a function of particle size and temperature [105]. Beyond quasi-static properties, atomistic spin dynamics has also been applied to thermally activated relaxation, enabling accurate parameterization of Arrhenius–Néel behavior (attempt frequency) for ${\mathrm{Fe}}_{3}$$O_{4}$ nanoparticles [106]. These results are important for interpreting magnetic hyperthermia and paleomagnetic stability, where relaxation times depend sensitively on anisotropy, size, and temperature.

Atomistic core–shell spin simulations have shown that surface vacancy-mediated pinning can be used to control low-temperature relaxation processes in γ-${\mathrm{Fe}}_{2}$$O_{3}$ nanoparticles [107]. At the mesoscale, interacting assemblies of maghemite nanoparticles can exhibit glassy magnetic regimes; a concentrated ensemble has been modeled and compared to canonical spin glasses, elucidating superspin-glass features and aging behavior [108]. These studies highlight that capturing maghemite magnetism often requires proper treatment of surface anisotropy, vacancy/disorder landscapes, and interparticle dipolar coupling.

### 5.4. Time and Length Scale

Time and length scale limitations further constrain predictive simulation capability. Ab initio MD is typically restricted to tens of picoseconds that are insufficient to capture all the features of a slow adsorption, restructuring and corrosion processes. Classical MD extends to nanoseconds, yet many technologically relevant processes occur over microsecond to millisecond scales. The next methodological milestone is routine nanosecond-microsecond sampling with near-DFT accuracy using machine learning potentials, enabling convergence of interfacial diffusion coefficients within 5% and adsorption residence times within 10% statistical uncertainty.

### 5.5. Predictive Interfacial Modeling

Ultimately, predictive interfacial modeling of iron oxides requires integrated multiscale frameworks capable of simultaneously describing adsorption thermodynamics, surface redox evolution, magnetic ordering, and electrochemical environment. Achieving quantitative reliability will depend on standardized benchmarking datasets, uncertainty quantification protocols, and transferable models validated across hematite, magnetite, and maghemite. These challenges are particularly relevant for applications such as hydrogen-, CO-, and carbon-assisted reduction of iron oxides, where interfacial reactions between oxide surfaces and reactive gas-phase or condensed-phase species govern oxygen removal, phase transformation, and metal extraction efficiency.

The predictive use of classical and reactive interatomic potentials also requires careful assessment of parameter transferability beyond the atomic environments used for fitting. This is particularly important for complex local structures such as defect cores, cation vacancies, grain boundaries, undercoordinated surface sites, and vacancy-rich maghemite configurations. In such cases, force field-based MD simulations may reproduce selected equilibrium bulk or surface properties while failing to capture local relaxation, defect energetics, charge redistribution, phase stability, or high-temperature dynamics with comparable reliability. Similar limitations can arise for reactive force fields such as ReaxFF, where predictions of bond breaking, oxidation-state changes, structural disordering, or melting behavior may depend sensitively on the reference data and fitting protocol. Therefore, when bulk defects, vacancy ordering, surface reconstruction, or thermal stability are central to the property of interest, validation against experimental measurements and/or electronic-structure benchmarks remains essential.

A key requirement for such frameworks is the consistent coupling of adsorption free energies, charge-transfer descriptors, defect/vacancy configurations, and magnetic or electrochemical boundary conditions within a common validation strategy.

The convergence of machine learning potentials, enhanced sampling, and electrochemical boundary conditions provides a credible pathway toward genuinely predictive, and magnetically aware simulations of iron-oxide interfaces.

### 5.6. Perspectives

The synergistic integration of machine learning interatomic potentials, spin-lattice dynamics, and electrochemically explicit MD is expected to transform iron-oxide modeling from descriptive to predictive science. To make these trends and remaining gaps more transparent, Table 4 provides a qualitative overview of the current state of evidence for each oxide and facet across classical MD, ReaxFF, AIMD, and machine learning interatomic potentials. Although the main facet comparison in this review focuses on (111) and (110) surfaces, ${\mathrm{Fe}}_{3}$$O_{4}$(001) is included because reconstructed magnetite (001) represents a major case in the atomistic simulation literature. Achieving quantitatively reliable adsorption free energies, magnetic transition temperatures, and redox energetics across hematite, magnetite, and maghemite will enable direct comparison with operando spectroscopy and microscopy experiments. A particularly important gap concerns facet-resolved MD of maghemite, where vacancy ordering and multiple metastable cation/vacancy configurations complicate the construction of transferable models and systematic slab comparisons. Such integrated frameworks will not only clarify facet-dependent surface chemistry on (111) and (110) surfaces but also provide atomistic design principles for corrosion-resistant coatings, catalytic interfaces, magnetic nanomaterials, and energy-storage systems. The next decade of research is thus likely to shift from isolated surface models toward fully coupled magnetic–electronic–chemical simulations capable of bridging atomistic insights to macroscopic functionality.

## 6. Conclusions

This review has summarized the state-of-the-art of MD simulations studies on hematite (α-${\mathrm{Fe}}_{2}$$O_{3}$), magnetite (${\mathrm{Fe}}_{3}$$O_{4}$), and maghemite (γ-${\mathrm{Fe}}_{2}$$O_{3}$), with emphasis on the crystal structure, facet-dependent surface chemistry, magnetic ordering, and adsorption of small molecules. Across these iron oxide phase, MD has evolved from rigid classical descriptions of bulk lattices to increasingly sophisticated, interface-aware and magnetically informed simulations capable of addressing adsorption thermodynamics, hydration structure, defect dynamics, and nanoparticle behavior.

Classical force fields, including Buckingham-type models, CLAYFF, and INTERFACE-FF, remain by far the energetic models of choice for long-timescale MD simulations for examining water structure, ion adsorption, and organic-surface interactions, particularly on magnetite (111) and hematite (110). Reactive force fields extend these capabilities toward redox chemistry and corrosion processes, while ab initio MD provides essential benchmarks for water dissociation, interfacial dynamics, and DFT-based interpretations of charge transfer and electronic polarization. More recently, machine learning interatomic potentials have emerged as a transformative approach, enabling near-DFT accuracy at nanosecond timescales and opening the door to statistically converged adsorption free energies and defect energetics.

Despite the great progress in atomistic simulations many significant challenges remain unresolved. Among others, mixed valence in magnetite, vacancy ordering in maghemite, and polaron formation in hematite still require the development of transferable interatomic potentials and electronic structure-based reference models. The explicit coupling between surface chemistry and magnetic degrees of freedom—particularly spin canting, anisotropy, and temperature-driven transitions such as the Morin transition—remains largely unexplored within adsorption-focused MD frameworks. Moreover, systematic comparisons between compact (111) and open (110) facets across all three oxides are still limited, especially under realistic aqueous and electrochemical conditions. Future advances will likely arise from integrated multiscale strategies that combine DFT+*U* datasets, machine learning potentials, enhanced sampling techniques, and spin-lattice dynamics.

The implementation of accurate interatomic potentials, spin-aware models, and electrochemical boundary conditions will allow for predictable simulations of iron oxide interfaces, facilitating the design of advanced materials and energy systems.

## Figures and Tables

**Table 1:** Summary of characteristic properties of the crystallographic structure of Iron Oxides [1].

Property	Hematite (α-${\mathrm{Fe}}_{2}$$O_{3}$)	Magnetite (${\mathrm{Fe}}_{3}$$O_{4}$)	Maghemite (γ-${\mathrm{Fe}}_{2}$$O_{3}$)
Structure type	Corundum	Inverse spinel	Defective spinel
Crystal system	Trigonal (rhombohedral)	Cubic	Cubic
Space group	R$\bar{3}$c	Fd$\bar{3}$m	${P4}_{3}$32 or Fd$\bar{3}$m
Lattice parameters (Å)	a=5.035, c=13.75	a=8.396	a=8.33–8.35
Oxygen sublattice	hcp-like	fcc	fcc
Fe coordination	Octahedral (${\mathrm{Fe}}^{3+}$)	${\mathrm{Fe}}^{3+}$ (tetrahedral + octahedral), ${\mathrm{Fe}}^{2+}$ (octahedral)	${\mathrm{Fe}}^{3+}$ (tetrahedral + octahedral)
Bulk Fe charge (*e*)	+3	+2/+3	+3
Bulk O charge (*e*)	−2	−2	−2

**Table 2:** Comparison of atomistic simulation methodologies applied to hematite, magnetite, and maghemite. System sizes and timescales are typical orders of magnitude.

Potential	Physics Captured	Typical System Size/Timescale
Buckingham, Coulombic [2]	Ionic bonding, lattice relaxation, diffusion, adsorption (fixed charges)	$10^{4}$–$10^{6}$ atoms; ns–μs
Core-shell (polarizable) [2,55]	Ionic bonding + electronic polarization (dielectric response, phonons)	$10^{3}$–$10^{5}$ atoms; ns
Reactive (ReaxFF) [3,56]	Bond breaking/formation, redox chemistry, charge equilibration	$10^{3}$–$10^{5}$ atoms; 100 ps–10 ns
AIMD (DFT, DFT+*U*) [68,69]	Explicit electronic structure, proton transfer, redox processes, magnetism (spin-DFT)	$10^{2}$–$10^{3}$ atoms; 10–100 ps
Spin-lattice dynamics [87,88]	Coupled atomic motion and classical spin dynamics; magnetoelastic effects	$10^{4}$–$10^{6}$ atoms; ns
Machine learning [77,78,79,80]	Near-DFT accuracy; scalable bond rearrangements if trained appropriately	$10^{4}$–$10^{6}$ atoms; ns–μs

**Table 3:** Summary of MD studies on adsorption of small molecules on hematite (α-${\mathrm{Fe}}_{2}$$O_{3}$), magnetite (${\mathrm{Fe}}_{3}$$O_{4}$), and maghemite (γ-${\mathrm{Fe}}_{2}$$O_{3}$) surfaces. The evidence status distinguishes conclusions supported by explicit MD/AIMD/ML-MD simulations from qualitative trends, DFT-based interpretations, or expected behavior where direct facet-resolved MD evidence remains limited.

Oxide	Facet	Water Adsorption (MD)	Nitrogen-Containing Molecules	Organic Molecules	Evidence Status	Representative MD References
Hematite	(111)	Ordered hydration layers; ML-MD resolves nanosecond interfacial dynamics	Sparse MD datasets	Organic adsorption sensitive to surface polarization	Explicit ML-MD evidence for water; limited or interpretive evidence for other adsorbates	[89,91,100]
Hematite	(110)	Strong interfacial layering; facet-controlled H-bond network; field-induced dissociation (AIMD)	Limited direct MD; higher site heterogeneity expected	Phenol adsorption influenced by solvent structuring; reactive MD shows polarity effects	Mixed: explicit AIMD/MD evidence and expected trends	[16,47,71,99,100]
Magnetite	(111)	Stabilized adlayers; hydroxyl formation; validated oxide–water force fields; ML-MD coverage studies	${\mathrm{NH}}_{3}$ and amines bind via Fe coordination; temperature-dependent adsorption	Oleic acid and formate show facet-dependent ordering; phenol adsorption mediated by hydration	Explicit MD-supported findings	[19,20,92,96,97,98]
Magnetite	(110)	Greater adsorption heterogeneity expected; limited explicit post-2020 MD comparisons	Potentially stronger Lewis-acid interactions (limited MD data)	Systematic organic adsorption MD scarce	Mostly expected trends; limited direct MD evidence	[17,93]
Maghemite	(111)	Vacancy-modulated hydration; electronic polarization effects (DFT-MD)	Very limited facet-resolved MD	Dye adsorption modeled on nanoparticle/composite systems	Limited direct MD evidence; partly DFT-MD or nanoparticle-based evidence	[76,101]

**Table 4:** Qualitative summary of the current state of atomistic simulation evidence for facet-resolved iron oxide surface modeling. Entries indicate whether evidence is relatively mature, limited, emerging, or currently underdeveloped for classical MD, ReaxFF, AIMD, and machine learning interatomic potentials.

Oxide	Facet	Classical MD	ReaxFF	AIMD	ML Potentials
Hematite	(111)	Established for hydration and interface structure	Limited for direct facet-resolved adsorption	Available for water/proton transfer and electronic interpretation	Emerging for hematite–water interfaces
Hematite	(110)	Established for hydration and surface-water structure	Available in selected reactive/slab studies	Available for water dissociation and field-driven chemistry	Limited but developing
Magnetite	(111)	Established for water, ammonia, amines, and organic adsorption	Relevant mainly through oxidation/reduction studies	Available for hydrothermal water interaction and redox processes	Emerging
Magnetite	(110)	Limited direct facet-resolved evidence	Limited direct evidence	Sparse	Underdeveloped
Magnetite	(001)	Important and well represented in reconstructed-surface studies	Available in selected reactive contexts	Available for reconstruction, water interaction, and ligand effects	Emerging, including neural network potential studies
Maghemite	(111)	Limited; vacancy effects remain challenging	Sparse	Available mainly through DFT-MD or nanoparticle/thin-film models	Underdeveloped
Maghemite	(110)	Very limited or absent direct MD evidence	Sparse	Sparse	Underdeveloped

## Data Availability

No new data were created or analyzed in this study. Data sharing is not applicable to this article.
